# From the Genetics of Ankylosing Spondylitis to New Biology and Drug Target Discovery

**DOI:** 10.3389/fimmu.2021.624632

**Published:** 2021-02-17

**Authors:** Zaarour Nancy, Li Yan, Shi Hui, Bowness Paul, Chen Liye

**Affiliations:** ^1^Nuffield Department of Orthopaedics, Rheumatology and Musculoskeletal Sciences, University of Oxford, Oxford, United Kingdom; ^2^Department of Rheumatology, The First Affiliated Hospital of Xiamen University, Medical College of Xiamen University, Xiamen, China

**Keywords:** ankylosing spondylitis, GWAS, functional genomics, IL-23/IL-17 axis, drug target, IL-1beta, genetics

## Abstract

Genome-wide association studies (GWAS) have identified 113 single nucleotide polymorphisms (SNPs) affecting the risk of developing ankylosing spondylitis (AS), and an on-going GWAS study will likely identify 100+ new risk loci. The translation of genetic findings to novel disease biology and treatments has been difficult due to the following challenges: (1) difficulties in determining the causal genes regulated by disease-associated SNPs, (2) difficulties in determining the relevant cell-type(s) that causal genes exhibit their function(s), (3) difficulties in determining appropriate cellular contexts to interrogate the functional role of causal genes in disease biology. This review will discuss recent progress and unanswered questions with a focus on these challenges. Additionally, we will review the investigation of biology and the development of drugs related to the IL-23/IL-17 pathway, which has been partially driven by the AS genetics, and discuss what can be learned from these studies for the future functional and translational study of AS-associated genes.

## Introduction

Ankylosing Spondylitis (AS) is a common form of immune-mediated arthritis that predominantly affects the sacroiliac and spinal joints and can result in excessive ossification of the affected tissues. Over the past decade the successful introduction of new treatments for AS (therapeutic monoclonal antibodies targeting tumor necrosis factor (TNF)-α and interleukin (IL)-17A) has highlighted some of the important pathological pathways involved. However, <50% of patients achieve good response (ASAS40) to either TNF-α or IL-17A blockade (1, 2). More importantly, there is no cure for AS and most patients require lifelong medication (with consequent potential adverse effects) to control their symptoms. Therefore, identifying novel therapeutic targets could have important benefits for patients with AS.

The value of genetics in drug discovery is increasingly appreciated (3, 4). The induction of IL-17A blockade in AS was partially driven by genetic studies showing multiple disease associations with genes involved in IL-23/IL-17A pathways (e.g., IL6R, IL23R, TYK2, IL1R1/2, IL27, STAT3) (5). Genome-wide association studies (GWAS) have already identified 113 single nucleotide polymorphisms (SNPs) affecting the risk of developing AS (6, 7). To date, there is a plausible explanation for only a minority of these genetic associations, substantially impeding their translation into therapeutic options.

The functional investigation of genetics association currently encountered a number of challenges: (1) difficulties in determining the causal genes regulated by disease-associated SNPs, (2) difficulties in determining the relevant cell-type(s) that causal genes exhibit their function(s), (3) difficulties in determining appropriate cellular contexts to interrogate the functional role of causal genes in disease biology. This review will discuss recent progress and remaining challenges. While appreciating the importance of identifying causal SNPs, limited by the length of this mini-review, we choose to refer readers to recent review rather than discuss this topic here (8). Following the identification of causal genes and related cellular contexts, immunological research is vital for drug discovery. We will use the IL-23/IL-17 pathway as an exemplar, in part driven by the AS genetics, and discuss what can be learned from these studies for the future functional and translational study of AS-associated genes.

## AS Genetics

Genetic contribution to the development of AS was first known following the discovery of HLA-B^*^27 as a strong genetic risk factor in 1973 (9–11). In fact, the association was so strong that HLA-B^*^27 was, for a long time, considered to be the sole genetic factor predisposing individuals to AS. Till 2007, powered by the technical development in SNP genotyping and statistical analysis for GWAS, the first AS GWAS was competed (12). Although with a relatively small sample size (1,000 patients and 1,500 controls), this study identified two key non-MHC genetic risks: IL23R and ERAP1. These findings were subsequently confirmed in a study with a larger cohort, which reported two additional associations with chromosome 2p15 and 21q22 (13). In the same year, a study focusing on 53 known genetic risks in Crohn's disease, a condition clinically related to AS, identified two additional AS-associated loci: 1q32 and STAT3 (14). In 2011, the striking epistasis between ERAP1 and HLA-B^*^27 was found, along with seven additional genetic loci with strong associations with AS (15). The most recent findings were reported from the Immunochip project with the strategy of high-density genotyping of immune-related loci, which, in part using “multi disease” methodology, has increased the number of SNPs independently affecting the risk of developing AS to 113 (6, 7).

Overall, a significant body of knowledge of AS genetics has been generated over the last decade. This rich and high-quality source of genetic risk associations in AS will, after appropriate decoding, provide critical sights in AS biology and new drug targets.

## Translating Genetics to New Biology and Drug Target Discovery

### Recent Technical Advances and Opportunities

**Figure 1:**
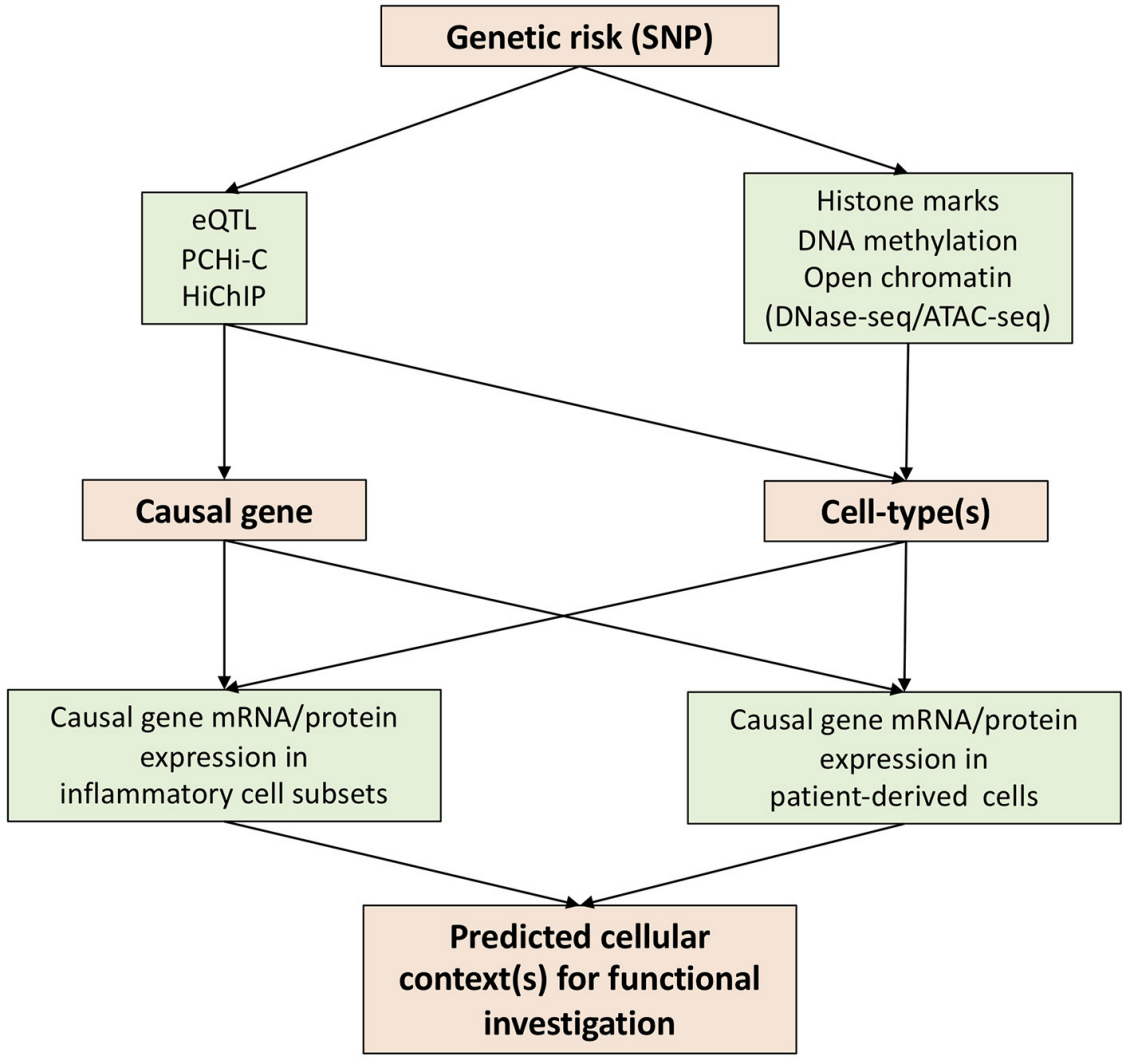
Strategies to translate genetic risk to novel biology.

In attempting to reveal the functional basis of genetic risks associated with human diseases, various techniques have been developed over the past few years. We believe that expression quantitative trait loci (eQTL), promoter capture Hi-C (PCHi-C), and HiChIP constitute key advances for the prediction of causal genes through the annotation of genetic risks (Figure 1).

Expression quantitative trait loci (eQTL) identifies genomic variants that contribute to altered expression levels of mRNAs. eQTL have been carried out using various primary human immune cells (monocyte, macrophage, dendritic cell, CD4, CD8, Treg, Th1, Th2, Th17, Tfh, B-cell, NK and neutrophil) in different cellular contexts (resting and activation) (16–21). These data constitute a rich eQTL data resource which can be integrated with summary data from AS GWAS studies for the prediction of the causal genes (22, 23). Of note, eQTLs are only present in a proportion of GWAS SNPs (24, 25), highlighting the need for additional approaches to link SNP to gene.

The development of chromosome conformation capture (3C) and its related techniques, such as Hi-C, has allowed the detection of long-range regulatory DNA interactions (26, 27). To overcome the nature of complexity and high-cost of Hi-C, promoter capture Hi-C (PCHi-C) has been developed, combining Hi-C with hybridization-based capture of targeted genomic regions (28). PCHi-C has been carried out for various diseases using relevant tissues/organs and/or cells (29–31), but not yet in AS. One dataset, which we believe will be of particular value for AS research, provides high-resolution maps of promoter interactions at the genome-wide level in 17 human primary blood immune cell types (32). HiChIP is another technique derived from Hi-C which incorporates ChIP-seq—allowing the enrichment of chromatin looping events based on histone modifications (33). H3K27ac HiChIP has been applied to naïve CD4, Th17, and Treg cells to reveal T cell subtype-specific enhancer–promoter interactions (34). These enhancers often contact genes beyond their nearest neighbor gene–highlighting the importance of SNP annotation using functional genomic datasets. Thus, we believe that the integration of chromatin looping datasets and AS GWAS findings provides a potent approach to predict the causal genes.

Determination of *disease-relevant* cell-types for functional investigation is a key challenge impeding the translation of genetic findings to new biology and therapeutic options. Some causal genes identified by eQTL or chromatin looping datasets will be limited in their action to specific cell-type(s), guiding the selection of cells to be investigated (Figure 1). However, this information is not always available. In such a scenario interrogation of chromatin accessibility (DNase hypersensitivity assay or ATAC-seq), DNA methylation and histone modifications will be of great use following mapping with GWAS SNPs (Figure 1). The latter include enhancer (e.g., H3K4me1), promoter (H3K4me3), and active enhancer and promoter marks (H3K27ac).

Precise functional testing of predicted causal genes requires knowledge of cellular context(s). This is particularly important for genes where existing knowledge of function is limited, a common situation for GWAS hits. To this end, transcriptional data from patient-derived cells and/or particular disease-related cell-types, such as Th17 cells in AS, would be of great use. For example, if a causal gene is elevated in Th17 cells, one would predict it to be a possible Th17 regulator and test its function in a Th17 functional cellular assay. Single cell RNA sequencing (scRNA-seq) would excel here in the provision of gene abundancy data for multiple cell subsets in patient blood or synovium. However, a well-known drawback of scRNA-seq is its inability to detect genes in low abundance. Antibody (CITE-seq) or oligo (BD Rhapsody)–based tagging of genes of interest might go some way to solve this problem. In addition, for genes with available antibodies, mass cytometry or CyTOF is an alternative approach to acquire the expression profile of a gene at the protein level.

### Remaining Challenges and Possible Solutions

eQTLs are frequently different in different cell types. For example, the eQTL link of GAB2 gene with rs2511162 is found for naïve B cells and T cells, but not monocytes (19). Even within one cell-type, eQTLs can be highly context-specific. For example the AA genotype at rs1179625 is associated with higher basal mRNA HIP1 levels in naïve monocytes, but reduced HIP1 upregulation in lipopolysaccharides (LPS)-stimulated monocytes (16). Of note, the difference in context is not limited to resting vs. stimulation but may be highly time dependent for a cell-type treated with the same stimuli. For examples, the eQTL linking rs2275888 with IFNB1 gene transcription is present in monocytes after 2 h LPS-stimulation but not in resting monocytes or those cultured with LPS for 16 h (16). As mentioned in the previous section, cell-type and context specificity are also present in chromatin looping datasets (PCHi-C and HiChIP). Thus, although current eQTL and chromatin looping datasets have included various conditions for an individual cell-type, they cannot possibly cover all the complex and dynamic microenvironments present in human diseases including AS. Given the high probability of the presence of AS-specific genetic regulations, this knowledge will be crucial in advancing our understanding of the impact of genetic risks on AS biology and unraveling novel mechanisms and therapeutic options. To this end, we propose that functional genomics datasets should ideally be generated using cells from blood or even joint of patients with AS for the provision of disease-specific insights.

Evidence suggesting key roles for rare immune cell populations in AS has recently emerged,. For example invariant NK cells (iNKT) and γδ T cells have recently been reported to be a major source of IL-17 in the inflamed joint (35). These innate-like T cells are phenotypically and functionally different from conventional T cells, thus would likely have distinct gene expression mechanisms. Neither eQTL nor chromatin looping datasets have been generated for these un-conventional cell types, and we propose that coordinated efforts to generate functional genomic datasets for these cells should be made by the scientific community.

Even within one cell-type, specific subsets might be highly relevant to the pathogenesis of human diseases. For example, using single cell RNA sequencing (scRNA-seq), MerTK+ synovial tissue macrophages have recently been shown to be key for the remission of rheumatoid arthritis after treatment cessation (36). Thus, scRNA-seq-based eQTL studies carried out using patient blood and/or tissue derived cells will be of great value. This approach was first reported in 2018 for a small cohort of 45 healthy donors (37). More recently, the single-cell eQTLGen consortium has been established and will provide standardized pipelines and guidelines for single-cell population genetics studies (38).

Most functional genomics data are at the DNA or RNA level. This does not invariably relate to cellular and cell surface protein expression. Advances in quantitative MS might allow QTL at the protein level. Indeed, quantitative proteomics has been utilized to advance knowledge in biology, such as the dynamic protein landscape of human Th17 differentiation (39), and the underlying mechanism of Myc controlling T cell proteomes and metabolic pathways (40).

It will also be important to contextualize the anatomical location of immune cells and their detailed functional interactions. The human tissue atlas will provide a framework and detailed spatial transcriptomic and protein expression studies of diseased tissue including entheses will undoubtedly enrich current knowledge. Without doubt the greatest knowledge gains will flow from the study of cells from inflamed tissues. We believe that obtaining these from human diseased tissues will be more informative given the limitations of current animal models and the rapid advances in single cell technology.

Moving from tissue level understanding to whole organism will be a further challenge. Animal models of AS have proved useful for studying specific pathogenic processes and offer opportunities for intervention. The HLA-B27 transgenic rat and the SKG mouse have both provided key insights, with the former model confirming the role of HLA B27, myeloid cells and gut flora in disease and the latter confirming the key role for the IL-23-17 pathway (see below). Considering both animal models and human studies it will also be important to distinguish the relative roles of tissue-resident and tissue-specific cells from those of circulating cells. We believe that using animal models to label leucocytes present in the gut mucosa (e.g., with photobleaching or fate mapping) and then follow their potential movement to joints and other inflammatory sites is likely to offer major insights into disease pathogenesis. Ultimately human experimental medicine studies will prove the key arbiters of target selection and will provide a rich source of data.

## The IL-23/IL-17 Pathway and AS

### IL-23/IL-17 Pathway

IL-23 is formed by P19 and P40 subunits with the later, along with P35, also forming IL-12 (41). IL-23 signals through the IL-23 receptor composed of IL-23R and IL-12Rβ1. IL-12 drives the differentiation of Th1 cells, whereas IL-23 is crucial for the survival and expansion of Th17 cells and can induce IL-17 production in memory T cells (42, 43). Additionally, IL-23 also induces IL-17 production by γδ T, NKT and innate lymphoid cells (44–46). In line with this, murine models support the T cell-mediated pathogenic role of IL-23 in inflammation in multiple organs, including joints, gut, brain (47–49). Of note, both IL-23 and IL-17A are required for the development of Spondyloarthritis-like pathology in SKG mice, a T-cell driven AS model with inflammation in arthritis, enthesitis, and ileitis (50).

### Relevance to AS Genetics

More than 90% of genetic risk SNPs are present in non-coding regions. Thus, IL23R, where genetic risk loci reside both within coding (the cytoplasmic tail) and non-coding regulatory regions, represents the exception rather than the norm. The genetic association of IL23R loci with AS was first reported in 2007 (12), the first elucidated being a coding change SNP, rs11209026, associated with Arg or Gln at position 381 of IL-23R protein. Interestingly, the same SNP also affects the risk of developing inflammatory bowel disease (IBD) (51), a condition closely linked to AS. Indeed, a subgroup of patients with AS develops IBD and the sub-clinical gut inflammation has been reported in over 60% of patients with AS (52). The same SNP is also associated with psoriasis, another condition closely linked to AS. The protective variant R381Q is associated with reduced function of IL-23R and Th17 response in both CD4 and CD8 cells (53).

### Pre-clinical/Clinical Development of Inhibitors Targeting IL-23/IL-17 Pathway

Antibodies blocking cytokines or receptors related to this pathway have been extensively tested in AS. IL-17A blockers have demonstrated efficacy and been approved for the treatment of AS (54, 55). In contrast, IL-23 inhibitors either targeting P40 or P19, have failed to show efficacy in clinical trials (56, 57). These results were unexpected considering the efficacy of IL-23 blockers for Crohn's disease, Psoriasis and psoriatic arthritis, conditions related to AS and with IL23R as a genetic risk (58–61). Of note, IL-17 inhibition was ineffective in Crohn's disease (62), suggesting the IL-23 biology beyond the simple induction of IL-17 cytokine secretion.

**Figure 2:**
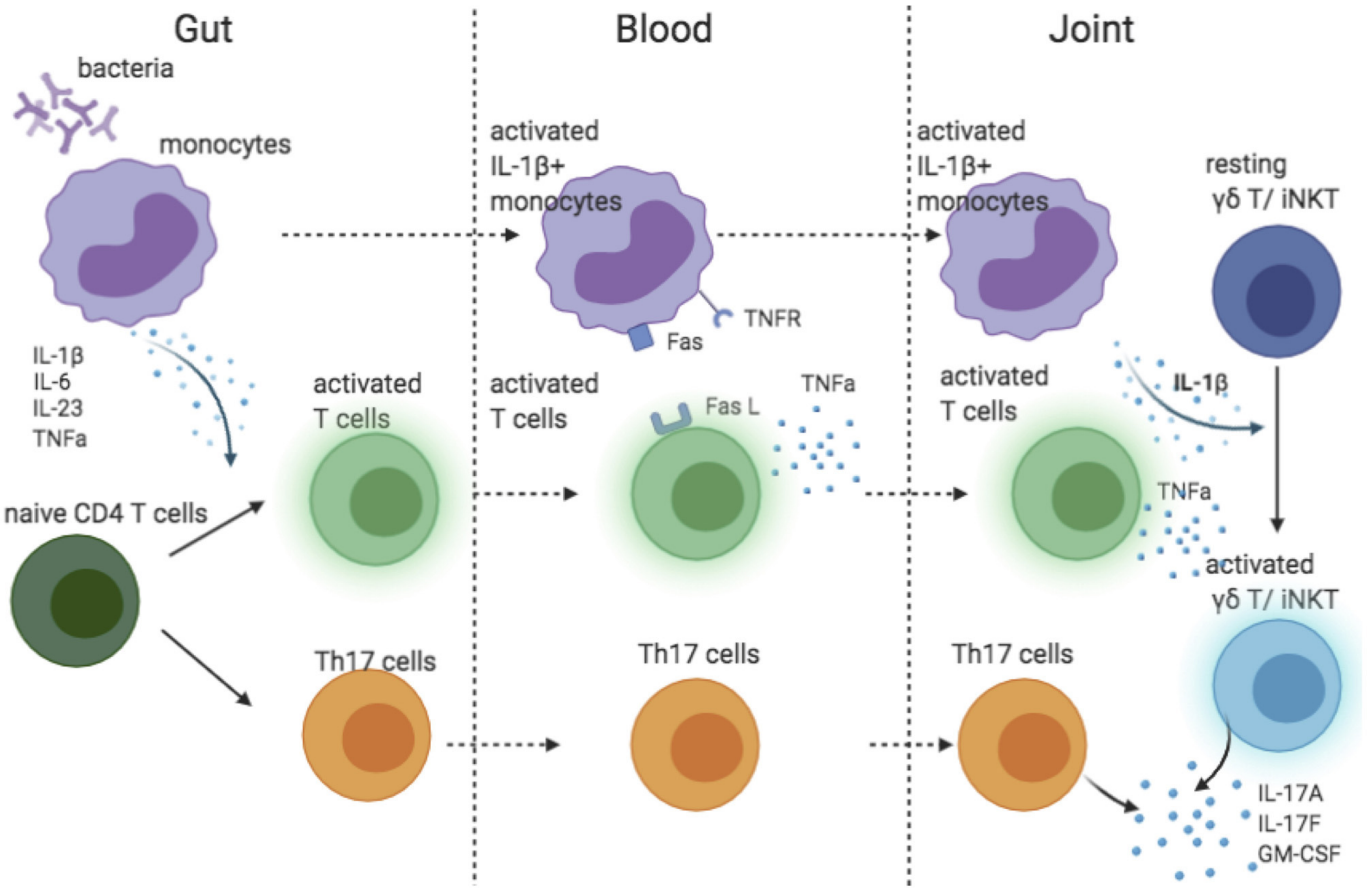
Cartoon model suggesting possible role of IL-1 beta contributing to IL-17 response in AS.

The success of IL-17A blocking and failure of IL-23 inhibition in AS suggested that IL-23 might not be the main driver of IL-17A production in AS. In human, IL-1β and IL-6 are required for the differentiation of Th17 cells (63). Of interest, IL-1β was essential in pathogen-induced Th17 differentiation to prime IL-17+IFN-γ+ “pathogenic” Th17 cells (64). Additionally, along with IL-23, IL-1β induces IL-17A production by γδ T and iNKT cells (45, 65), the major source of IL-17A in synovial fluid of patient with AS (35). The recruitment of IL-1β-producing myeloid cells has been shown to be a key factor driving the IL-17 secretion by γδ T and CD4 cells in the central nervous system (66). Two pieces of evidence link IL-1β to AS pathology: (1) both IL1R1 and IL1R2 are predicted genetic risks in AS (13), (2) monocytes in blood from patients with AS spontaneously produce IL-1β (67). Thus, we propose a model explaining the possible IL-1β-driven IL-17 biology in AS (Figure 2). Monocytes stimulated by bacteria in the gut produce pro-inflammatory cytokines that prime Th17 cells. Attracted by chemokines, IL-1β-secreting monocytes travel to the joint(s), where they activate γδ T and iNKT cells. Additionally, through a TNFR-Fas-caspase-8-dependent pathway, activated T cells also induce monocyte IL-1β secretion (68). However, IL-1β is unlikely to be the sole driver of IL-17 in AS because IL-1β inhibition was only effective for a subgroup of patients (69–71).

### Lessons From IL-23/IL17

The therapeutic development of inhibitors targeting the IL-23/IL-17 pathway in AS highlights the notion that genetic risk alone is not necessarily the ideal guide to drug target identification and that downstream protein(s) might be better therapeutic options in some cases. Indeed the association of genetic risk with drug success in trails is substantially enhanced when proteins interacting with these risk-associated gene products are included (72). Considering the diseases that share IL23R risk associations, significant differences in therapeutic response to different agents have already emerged. The reasons why IL-23 neutralization proved highly beneficial in psoriasis but without efficacy (at least in initial trials) in Ankylosing Spondylitis, whereas IL-17 neutralization proved therapeutic in Psoriasis, psoriatic arthritis and ankylosing Spondylitis but not Crohn's disease have been discussed by Siebert and colleagues (73). Thus, it is increasingly clear that, following the identification of the causal genes, detailed understanding of the biological functions of the associated proteins in the context of both tissue site and stage of disease is crucial.

## Discussion

Exciting progress has been made in the genetics of AS, resulting in identification of over one hundred genetic variants that affect the risk of disease development. Entering the post-GWAS era, we have encountered multiple challenges and bottlenecks in the translation of GWAS findings to new biology and drug targets. With the rapid development of functional genomic techniques/methods and transcriptomic and phenotypic profiling of primary cells at single cell resolution, it is now possible to predict both causal genes and their relevant cell-type. This will allow us to more rigorously investigate the cellular contexts of disease pathogenesis and to functionally validate therapeutic targets. However, disease-specific functional genomic datasets and those for rarer immune cells are currently not available, representing opportunities for future research. The successful development of drugs targeting the IL-23/IL-17 axis for conditions genetically associated with IL23R is a great example demonstrating the value of genetics in drug development. We also learned that causal genes are not always the best drug targets, highlighting the importance of establishing downstream pathways. Thus, an in-depth understanding of causal gene-related biology is absolutely crucial for the development of novel treatment options.

## Author Contributions

All authors listed have made a substantial, direct and intellectual contribution to the work, and approved it for publication.

## Conflict of Interest

The authors declare that the research was conducted in the absence of any commercial or financial relationships that could be construed as a potential conflict of interest.
